# Intercellular cross-talk through lineage-specific gap junction of cancer-associated fibroblasts related to stromal fibrosis and prognosis

**DOI:** 10.1038/s41598-023-40957-1

**Published:** 2023-08-30

**Authors:** Seong Ju Cho, Ji-Hye Oh, Jaehoon Baek, Yunsu Shin, Wonkyung Kim, Junsu Ko, Eunsung Jun, Dakeun Lee, Seok-Hyung Kim, Insuk Sohn, Chang Ohk Sung

**Affiliations:** 1 Department of Life Sciences, Pohang University of Science and Technology (POSTECH), Pohang, Republic of Korea; 2 Arontier Inc., Gangnam-Daero 241, Seocho-Gu, Seoul, Republic of Korea; 3 Department of Pathology, Asan Medical Center, University of Ulsan College of Medicine, 88 Olymphic-Ro 43-Gil, Seoul, 05505 South Korea; 4 Department of Medical Science, Asan Medical Institute of Convergence Science and Technology, Asan Medical Center, University of Ulsan College of Medicine, Seoul, Republic of Korea; 5 Division of Hepatobiliary and Pancreatic Surgery, Department of Surgery, Ulsan University College of Medicine and Asan Medical Center, Seoul, Korea; 6 Department of Pathology, Ajou University School of Medicine, Suwon, South Korea; 7 Department of Biomedical Sciences, Ajou University Graduate School of Medicine, Suwon, South Korea; 8 Department of Pathology, Samsung Medical Center, Sungkyunkwan University School of Medicine, Seoul, 06351 Republic of Korea

**Keywords:** Cancer, Biomarkers

## Abstract

Stromal fibrosis in cancer is usually associated with poor prognosis and chemotherapy resistance. It is thought to be caused by fibroblasts; however, the exact mechanism is not yet well understood. The study aimed to identify lineage-specific cancer-associated fibroblast (CAF) subgroup and their associations with extracellular matrix remodeling and clinical significances in various tumor types using single-cell and bulk RNA sequencing data. Through unsupervised clustering, six subclusters of CAFs were identified, including a cluster with exclusively high gap junction protein beta-2 (GJB2) expression. This cluster was named GJB2-positive CAF. It was found to be a unique subgroup of terminally differentiated CAFs associated with collagen gene expression and extracellular matrix remodeling. GJB2-positive CAFs showed higher communication frequency with vascular endothelial cells and cancer cells than GJB2-negative CAFs. Moreover, GJB2 was poorly expressed in normal tissues, indicating that its expression is dependent on interaction with other cells, including vascular endothelial cells and cancer cells. Finally, the study investigated the clinical significance of GJB2 signature score for GJB2-positive CAFs in cancer and found a correlation with poor prognosis. These results suggest that GJB2-positive CAF is a unique fibroblast subtype involved in extracellular matrix remodeling, with significant clinical implications in cancer.

## Introduction

Desmoplastic stromal fibrosis is frequently observed in cancer tissues such as pancreatic cancer, and cancer-associated fibroblasts (CAFs) are known to play an important role in the fibrosis, promoting tumor progression, metastasis, and therapeutic resistance^1–4^. However, there is still a lack of biomarkers for CAF and formation of fibrosis is a complex process. Although it is known to be associated with cross-talk between CAFs and tumor cells, the detailed mechanism has not yet been fully elucidated^4^.

Recent studies have shown that CAFs are not a homogeneous cell population, but rather a heterogeneous group with distinct phenotypes and functions^5–8^. The subtypes of CAFs such as inflammatory CAF (iCAF) and myofibroblastic CAF (myCAF) have been identified^9^, but specific markers for CAFs have not yet been discovered, making it difficult to study targeted therapies or mechanisms for CAF. Therefore, identifying CAF subtypes and their characteristics is critical for understanding the tumor microenvironment and developing effective therapies. CAF, known to generally promote tumor growth and metastasis, has been considered a promising target for a long time^10^. However, most clinical trials targeting CAF have resulted in failures^11,12^. This could be attributed to the heterogeneity of CAF and their dynamic changes alongside tumor growth^11,12^. Therefore, it is crucial to consider this dynamic nature when studying CAF. Recent advancements in single cell sequencing technology have greatly aided in understanding the heterogeneity and plasticity of CAF.

In this study, we aimed to identify CAF lineage specific biomarker and to investigate their functions in the tumor microenvironment. Through single-cell RNA sequencing (scRNA-seq) analysis of fibroblasts from multiple tumor types, we identified a subgroup of CAFs that express high levels of gap junction protein beta-2 (GJB2), and we named this subgroup "GJB2-positive CAFs." We further explored the characteristics and functions of GJB2-positive CAFs in the tumor microenvironment, including their association with extracellular matrix (ECM) remodeling and collagen production, their interactions with other cells, and their clinical significance. Our study suggests the existence of specialized fibroblasts associated with stromal fibrosis formation with lineage specific gene expression and provides new insight into the importance of vascular endothelial cells in fibrosis.

## Materials

### Analysis of scRNA-seq datasets

Public scRNA-seq datasets were used for this study. Three colorectal cancer datasets^13–15^ (GSE132465, GSE144735, and GSE178341) and a skin squamous cell carcinoma dataset^16^ (GSE144236) were obtained from the Gene Expression Omnibus. Breast cancer and lung cancer datasets were obtained from the ArrayExpress database (E-MTAB-8107, E-MTAB-6149, and E-MTAB-6653)^15^. The pancreatic cancer dataset was obtained from the Genome Sequence Archive (CRA001160 under project PRJCA001063)^17^. To minimize bias, we exclusively collected datasets generated using the 10 × Genomics platform. Additional data for pancreatic ductal adenocarcinoma, non-small cell lung cancer, and fibroblasts from non-cancer tissues, including diopathic pulmonary fibrosis, coronavirus disease, and ulcerative colitis, were obtained from Buechler et al.^18^. The scRNA-seq data for fetal/embryo tissue were also obtained from ArrayExpress (E-MTAB-11343)^19^. The spatially resolved transcriptome analysis of colorectal cancer from the 10xVisium platform was performed using STOMICS DataBase (Dataset ID: STDS0000033)^20^. This study approved by the Institutional Review Board of Asan Medical Center, Seoul, South Korea.

### Filtration of raw count matrix and log-normalization

Filtration of the raw count matrix, normalization, scaling, dimensionality reduction, cell clustering, and differential gene expression analysis of the processed data were performed in the Seurat package (v 4.1.0)^21^ in R (v 4.1.2). The raw count matrix was converted to a Seurat object using the ‘CreateSeuratObject’ function. We filtered out low quality cells with ≤ 200 or ≥ 6000 detected genes, unique molecular identifier (UMI) counts ≤ 1000, or a mitochondrial gene ratio ≥ 20%. After filtration, we normalized the data in each sample using the ‘NormalizeData’ function with default parameters, which generated log(feature counts/total counts for that cell + 1) data.

### Dimensionality reduction and clustering analysis

To identify the top 1000 variable genes for dimensionality reduction, we used the ‘FindVariableFeatures’ function. We set the parameters as follows: dispersion cutoff > 0.5, 0.0125 < mean cutoff < 3. The expression levels of the genes were then scaled, and the ‘RunPCA’ function was applied to the scaled data (except for the CAFs) to achieve dimensionality reduction. For the CAFs in each discovery sample, we intersected variable genes across the discovery samples to obtain common variable features before applying the ‘RunPCA’ function. Using these common variable features for principal component analysis reduced outliers owing to sample-specific bias. The elbow of the principal component analysis scree plot was used to determine the number of principal components to use for clustering. To visualize the clustered result, we applied the Uniform Manifold Approximation and Projection algorithm. For the clustering of each dataset and the construction of a shared nearest neighbor graph, we used the ‘FindNeighbors’ function and the ‘FindClusters’ function with default parameters; the resolution and dimensionality were determined depending on each dataset.

### Cell cluster annotation

We used the ‘SingleR’ R package (v 1.8.1)^22^ for unbiased cell-type recognition with a human reference data set from the Human Primary Cell Atlas^22^. To verify the cell-type recognition, we compared the annotations with known cell-type-specific differentially expressed genes. For elaborate fibroblast annotations, we annotated the cells as fibroblasts if they satisfied both of the following conditions^5^: the cells were annotated as ‘Fibroblast’ by ‘SingleR’ and the cells expressed one or more fibroblast markers (*DCN, COL3A1,* or *THY1*)^5,13,15^. The three markers were found to be commonly expressed in various fibroblast subtypes of colorectal cancer^13^, and our previous study also confirmed a similar pattern in fibroblast subtypes of several other cancer types^5^. The fibroblasts and epithelial cells taken from cancer tissues were annotated as ‘CAF’ and ‘Cancer,’ respectively, while those taken from normal tissues were annotated as normal fibroblasts (‘NF’) and ‘epithelial cells,’ respectively. For the GSE144735 sample, the fibroblasts from the border tissue and cancer tissue were not distinguishable on the Uniform Manifold Approximation and Projection plot; therefore, we also annotated the fibroblasts from the border tissue as ‘CAF’.

### Finding differentially expressed genes and robust markers of the CAF subtypes

The differentially expressed genes of each cell cluster in the RNA assay were computed using the ‘FindAllMarkers’ and ‘FindMarkers’ functions. To find differentially expressed genes consistently across the discovery samples, we intersected the differentially expressed genes of the same cell types. Then, we calculated the mean p-values and fold-change values to use for further analysis. For the CAF subtypes specifically, we applied the ‘FindAllMarkers’ function to find robust markers across the discovery samples. The 75 genes with the lowest p-value in each subtype were intersected to find robust markers. If the sign of the average log2(fold-change) as not constant across the discovery samples, we removed the gene from the list of robust markers.

### CAF subtype classification

To classify the CAF subtypes consistently across the discovery samples, we performed canonical correlation analysis using the ‘cancor’ function in the ‘stats’ R base package. Canonical correlation analysis was performed between the clusters obtained from different discovery samples, and then repeated to all possible cluster combinations. The input of the ‘cancor’ function was a normalized count matrix; the rows were the common variable features and the columns were the cells in the cluster. We then compared the correlations between the first canonical variates of all possible combinations to identify the highest correlation. If one combination showed the highest correlation, we considered the clusters in that combination to be the same subtype. If two combinations showed the same correlation between the first canonical variates, we compared the correlations between next canonical variates, until a difference was observed. If the subtype had a definite marker, it was utilized to annotate the subtype.

### Pseudotime analysis

The ‘monocle’ (v 2.22.0)^23^ R package was used to construct a trajectory and find the pseudotime of the CAFs. The CellDataSet object was constructed by using the ‘newCellDataSet’ function on the ‘RNA’ assay data of the Seurat object. Then, we submitted the CellDataSet object to the ‘estimateSizeFacotrs’ and ‘estimateDispersions’ functions for further analysis. The ‘differentialGeneTest’ function was used to find the differentially expressed genes among the six CAF subtypes and NF, which were used in the psuedotime analysis. Only differentially expressed genes with adjusted p-values < 0.001 were submitted to the ‘setOrderingFilter’ function to construct a trajectory. We used the ‘reduceDimension’ function with the following parameters to reduce the dimensions in the monocle: max_components = 2 and method = ‘DDRTree’. Then, we calculated the Spearman correlations between pseudotime and the normalized count values for all the features. All the Spearman correlations used in this paper were performed in the ‘cor.test’ function in the ‘stats’ R base package. Next, we calculated the mean correlation between the discovery samples. We only used genes that were measured across all the discovery samples (22,340 genes).

### Inferring cell–cell interactions using CellChat

Cell–cell interactions were inferred by the CellChat (v 1.6.1) R package^24^. CellChat can quantitatively identify cell–cell interactions and cell–cell communication. We used the curated human database in CellChat and the cell annotations determined by SingleR. We applied CellChat to each discovery sample with default parameters. The cancer cells were divided into several clusters, and we used these cancer clusters as inputs to consider heterogeneity. T cells, macrophages, monocytes, and endothelial cells were used in the same way as the cancer cells. To explore the interactions between CAFs and other cell types, we extracted the cell–cell interactions which were saved in the ‘net’ slot by the ‘subsetCommunication’ function. We collected the overlapping interactions between the clusters and CAFs in each sample, and then selected common interactions between the samples.

### Measurement of positive cells

If a cell had a non-zero UMI count for a certain gene, we considered it a positive cell for that gene. Then, to identify CAF-specific genes, we counted the cells that were positive for all possible genes on the CAFs and NFs across the discovery samples.

### Pathway analysis

Gene set enrichment analysis **(**GSEA) was performed in the GSEAplot (v 0.1.0)^25^ R package. We compared GJB2-positive CAFs with GJB2-negative CAFs in the ‘hallmark gene set’ and ‘gene ontology biological process’ datasets from The Molecular Signatures Database. Specifically, we only used the gene ontology biological process gene sets if the gene set name contained ‘MESEN’ or ‘FIBRO’. To achieve consistent GSEA results across the discovery samples, we calculated the average normalized enrichment score and nominal p-value, then used average values when considering the GSEA result.

### Correlation between GJB2 expression and marker gene sets

To investigate the characteristics of GJB2, we calculated the Spearman correlations between the GJB2 expression value and the mean expression value of each marker gene set. The marker gene sets were sourced from the literature^14,26^ (Supplementary Table S1).

### Proportion of GJB2-positive CAFs according to tumor stage

To investigate if GJB2-positive CAFs are related to poor prognosis, the proportion of GJB2-positive CAFs was calculated according to tumor stage. We counted the number of cells in the GJB2-positive and GJB2-negative CAF clusters for each tumor stage in the discovery samples. To integrate the notation for the tumor stage, we categorized the stages as follows: Stage 1, Stage 2, and LOW became ‘low stage,’ while Stage 3, Stage 4, and HIGH became ‘high stage’ (GSE178341 uses ‘LOW’ and ‘HIGH’ in its metadata). Then, we calculated the proportions of the cells in the GJB2-positive CAF clusters, and the numbers of CAF cells at each categorized tumor stage.

### The Cancer Genome Atlas bulk tissue RNA-seq data and clinical information

Normalized gene expression data (illuminahiseq_rnaseqv2-RSEM_gene_normalized) and corresponding clinical data for 8469 cancer tissues were downloaded (https://gdac.broadinstitute.org/). The dataset included the following cancer types: bladder urothelial carcinoma (BLCA, *n* = 407), breast invasive carcinoma (BRCA, *n* = 1079), cervical and endocervical carcinoma (CESC, *n* = 294), colon adenocarcinoma (COAD, *n* = 274), esophageal carcinoma (ESCA, *n* = 181), glioblastoma (GBM, *n* = 151), head and neck squamous cell carcinoma (HNSC, *n* = 515), kidney renal clear cell carcinoma (KIRC, *n* = 510),

kidney renal papillary cell carcinoma (KIRP, *n* = 283), brain lower grade glioma (LGG, *n* = 513), liver hepatocellular carcinoma (LIHC, *n* = 366), lung adenocarcinoma (LUAD, *n* = 510), lung squamous cell carcinoma (LUSC, *n* = 484), ovarian serous cystadenocarcinoma (OV, *n* = 299), pancreatic adenocarcinoma (PAAD, *n* = 177), prostate adenocarcinoma (PRAD, *n* = 493), rectum adenocarcinoma (READ, *n* = 88), sarcoma (SARC, *n* = 253), skin cutaneous melanoma (SKCM, *n* = 76), stomach adenocarcinoma (STAD, *n* = 412), testicular germ cell tumors (TGCT, *n* = 133), thyroid carcinoma (THCA, *n* = 497), thymoma (THYM, *n* = 1,119), and uterine corpus endometrial carcinoma (UCEC, *n* = 355).

### Single-cell RNA-seq sample preparation, library preparation, and sequencing

CAFs were isolated from endoscopic ultrasound-guided biopsy samples from patients with pancreatic cancer and maintained in Dulbecco’s modified Eagle’s medium (HyClone Laboratories) supplemented with 10% FBS, 1% penicillin, and streptomycin. We used the CAFs within six passages to avoid potential senescence-associated phenotypic changes. The use of the CAFs was approved by the Institutional Review Board of Ajou University Hospital (AJIRB-BMR-SMP-20-222).

For scRNA-seq, the 10 × Genomics Chromium platform was used to capture and barcode the cells to generate single-cell gel beads-in-emulsion, according to the manufacturer’s protocol. Briefly, along with the reverse transcription master mix, cell suspensions were loaded onto 10 × Genomics Single Cell 30 Chips. During this step, the cells were partitioned into the gel beads-in-emulsion, along with gel beads coated with oligonucleotides. These oligonucleotides enable mRNA capture inside the droplets by 30-bp oligo-dT after cell lysis, and provide barcodes to index the cells (16 bp) and transcripts (12-bp UMI). Following reverse transcription, cDNAs with both barcodes were amplified, and a library was constructed using the Single Cell 3′ Reagent Kit (v3.1 chemistry) for each sample. The resulting libraries were sequenced on an Illumina NovaSeq 6000 System in 2 × 150-bp paired-end mode.

### Sample demultiplexing, barcode processing, and UMI counting

We performed sample demultiplexing, barcode processing, and UMI counting using the official 10 × Genomics pipeline Cell Ranger (v6.1.1) (https://support.10xgenomics.com). Briefly, the raw base call files generated by Illumina sequencers were demultiplexed into reads in the FASTQ format using the bcl2fastq conversion software developed by Illumina (https://github.com/brwnj/bcl2fastq). The raw reads were trimmed from the 3′ end to obtain the recommended number of cycles for the read pairs (Read 1: 28 bp; Read 2: 90 bp). The reads from each library were then processed separately using the ‘cellranger count’ pipeline to generate a gene–barcode matrix for each library. During this step, the reads were aligned to the human reference genome (GRCh38). Cell barcodes and UMIs associated with the aligned reads were subjected to correction and filtering, and the count matrix data were pre-processed using the Seurat R package (v4.1.1). UMIs with < 401, expressed genes > 6000 or < 200, and with > 20% of the read mapped to the mitochondrial RNA were filtered out. For visualization, we performed principal component analysis with 2000 highly variable genes for initial dimensionality reduction, using t-distributed stochastic neighbor embedding to reduce the principal component dimensions into a 2D space. The scRNA-seq data have been deposited in the Gene Expression Omnibus with accession No. GSE223858.

### Statistical analysis

Statistical analyses were performed using R version 4.2.1. Differences were compared using the two-tailed Mann–Whitney U test or t-test. Correlation analysis of the continuous variables was performed using Spearman or Pearson correlation analysis. Log-rank tests were performed to evaluate survival differences between groups. Multivariate Cox proportional-hazards regression analyses were also performed.

### Ethics approval and consent to participate

This study was approved by the Ethical Committee of Asan Medical Center. Informed consent was obtained from all participants and all methods were carried out in accordance with relevant guidelines and regulations.

## Results

### Subgroup identification of GJB2-expressing CAF

**Figure 1:**
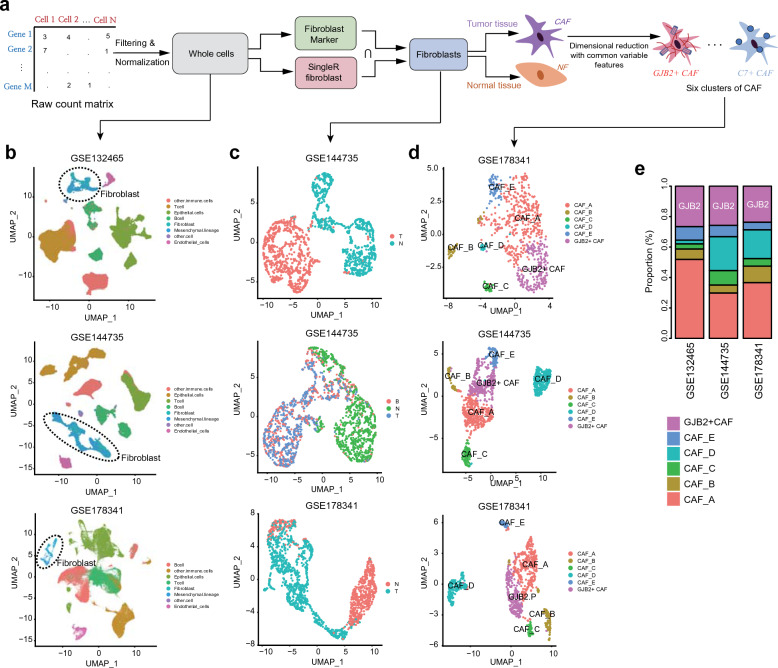
Identification of CAF subgroup. (**a**) Common fibroblast extraction from scRNA-seq data. (**b**) Clustering of all cell types in three colorectal cancer datasets. (**c**) Clustering of fibroblasts in cancer and normal tissues. (**d**) Clustering of CAFs (fibroblasts in tumor tissues) revealed six subgroups, which were consistent in three independent datasets. (**e**) GJB2-positive CAF is one of the major subgroups. *CAF* cancer-associated fibroblast.

To identify CAF subgroups, fibroblasts were extracted from the scRNA-seq data of multiple tumor types using the intersection of two independent approaches for robust fibroblast identification (Fig. 1a). Using whole cells, the identified fibroblasts were clustered into several groups (Fig. 1b). Among the fibroblasts, the CAFs and NFs clustered separately in multiple tumor types (Fig. 1c). When the CAFs were further divided using unsupervised clustering, a total of six subclusters were identified. Among these six subclusters, one showed exclusively high GJB2 expression (Fig. 1d and Supplementary Fig. S1a). We called this subcluster “GJB2-positive CAF” and, on average, it accounted for 25.3% of all the CAFs across the multiple datasets (Fig. 1e and Supplementary Fig. S1b). GJB2 expression is not specific to fibroblasts, as it is also observed in epithelial cells and immune cells (Supplementary Fig. S1c). Several subtypes of NFs showing pericyte-like, stem cell, inflammatory, or myogenic features were identified and these subtypes were similar across various datasets (Supplementary Fig. S2). The expression of GJB2 was almost negative even at the subtype level of these NFs (Supplementary Fig. S2).

### GJB2-positive CAFs are terminally differentiated and associated with collagen gene expression

**Figure 2:**
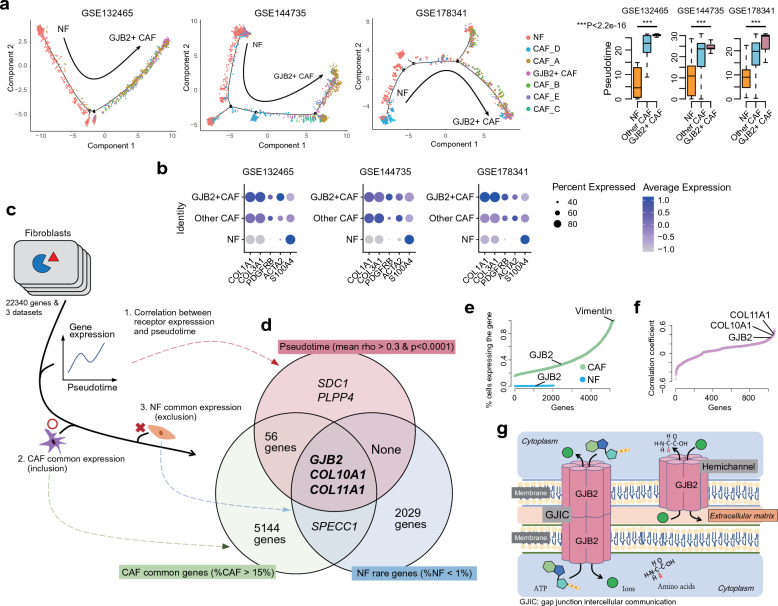
Molecular characteristics of GJB2-positive CAFs. (**a**) Trajectory analysis of fibroblasts showed that GJB2-positive CAFs are terminally differentiated. (**b**) The expression of proto-typical genes of CAF in fibroblast subgroups. (**c**) Identification of CAF-specific genes based on pseudotime and differentially expressed genes between CAF and NF. (**d**) GJB2 was identified as one of the most significant genes for CAF. (**e**) Gene expression pattern in CAF and NF. (**f**) Correlation between pseudotime and gene expression showing that GJB2 is expressed in terminally differentiated CAF. (**g**) Schematic illustration of GJB2 functions. *CAF* cancer-associated fibroblast, *NF* normal fibroblast.

Trajectory analysis using NFs and CAFs revealed that the GJB2-positive CAF subgroups were mostly located towards the end of the pseudotime trajectory, indicating that the GJB2-positive CAF group is a terminal differentiation stage in various NF and CAF subgroups (Fig. 2a and Supplementary Fig. S3). The proto-typical gene expressions including *COL1A1, COL3A1, PDGFRB, ACTA2,* and *S100A4* for CAF showed higher expression in CAF subgroups than NF (Fig. 2b). Meanwhile, to discover genes related to the differentiation of CAF, we evaluated gene expression patterns based on pseudotime, the inclusion of commonly expressed CAF genes, and the exclusion of commonly expressed NF genes (Fig. 2c and Supplementary Tables S2–S4). Using this approach, GJB2, COL10A1, and COL11A1 were identified (Fig. 2d). In the fraction of fibroblasts with gene expression (Fig. 2e), vimentin was expressed in most CAFs, irrespective of the CAF subgroup, whereas GJB2 was expressed in a CAF subgroup but seldom expressed in NF. When the gene expression was correlated with pseudotime, GJB2, COL10A1, and COL11A1 were top ranked (Fig. 2f). Overall, these findings suggest that GJB2-positive CAFs are a unique subgroup of terminally differentiated CAFs associated with collagen gene expression. Considering that GJB2 is known to have two major functions^27^, gap junction intercellular communication and hemichannel formation (Fig. 2g), and that GJB2-positive CAFs were significantly associated with collagen gene expression, we speculated that ECM remodeling through hemichannels could be a major function of GJB2-positive CAFs.

### GJB2-positive CAFs are associated with ECM remodeling

**Figure 3:**
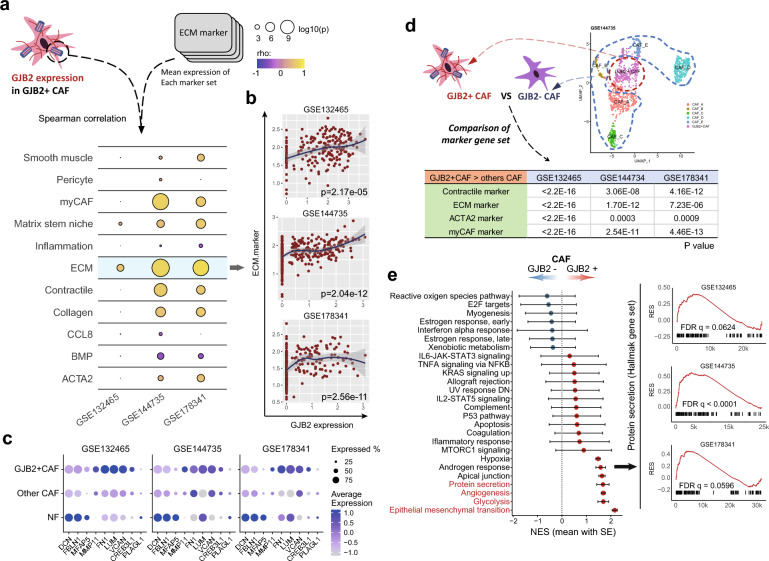
Functional ontology terms enriched in GJB2-positive CAFs. (**a**) Ontology terms associated with ECM were enriched in GJB2-positive CAFs. (**b**) ECM gene signature was correlated with GJB2 expression in CAFs (Spearman correlation test). (**c**) The expression of individual genes related with the ECM in fibroblast subgroups. (**d**) Differential gene ontology between GJB2-positive and GJB2-negative CAFs (Wilcoxon-rank sum test). (**e**) GSEA pathway analysis using hallmark gene set. *CAF* cancer-associated fibroblast, *ECM* extra cellular matrix.

Next, we further evaluated the function of GJB2-positive CAFs in cancer tissues. GJB2 expression in this CAFs was significantly correlated with gene expression in the ECM (Fig. 3a). This association was observed in all three datasets (Fig. 3b). The expression of individual genes associated with the ECM is higher in GJB2-positive CAFs compared to GJB2-negative CAFs (Fig. 3c). The overexpressed genes in GJB2-positive CAFs compared with GJB2-negative CAFs also indicated an association with the ECM (Fig. 3d). Moreover, pathway analysis using GSEA revealed enriched pathways of protein secretion, angiogenesis, and the ECM in GJB2-positive CAFs compared with GJB2-negative CAFs (Fig. 3e).

### Communication of GJB2-positive CAFs with vascular endothelial cells and cancer cells

**Figure 4:**
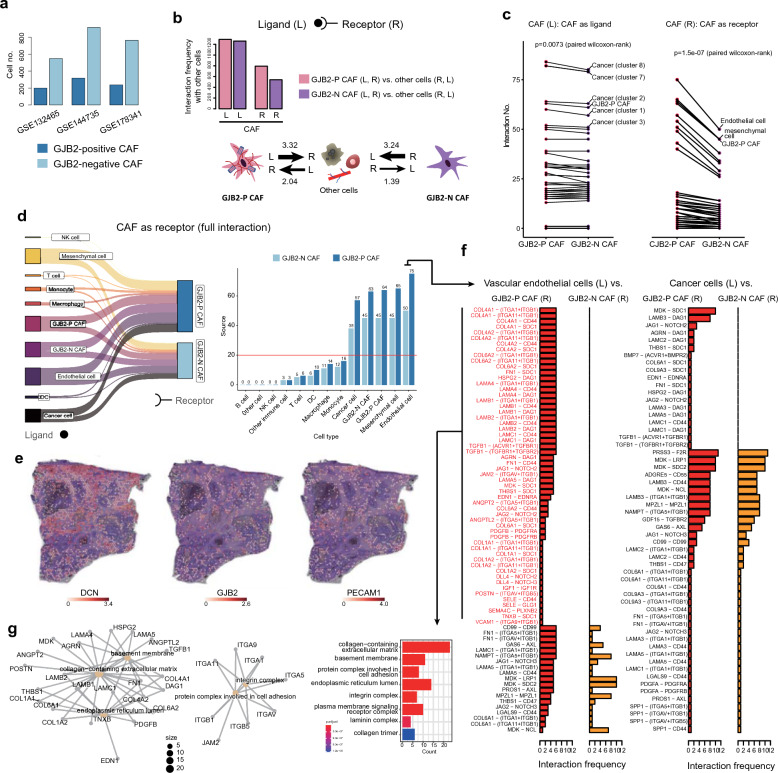
Cell–cell interactions of GJB2-positive CAFs and their expressions. (**a**) Cell fraction of GJB2-positive or GJB2-negative CAFs. (**b**) Cell interaction frequencies of GJB2-positive or GJB2-negative CAFs. (**c**) Higher interaction frequencies in GJB2-positive CAFs compared with GJB2-negative CAFs (paired Wilcoxon-rank sum test). Cancer cells and endothelial cells interact the most frequently with GJB2-positive CAFs. (**d**) Sankey plot for interactions between CAFs and other types of cells. (**e**) Gene expression of *DCN*, *GJB2*, and *PECAM1* (CD31) in spatially resolved transcriptome data. (**f**,**g**) Ligand–receptor pairs frequently identified in GJB2-positive CAFs and their function.

To identify the interactions between GJB2-positive CAFs and other cells, we analyzed ligand–receptor interactions using CellChat. The number of GJB2-negative CAFs were higher than the number of GJB2-positive CAFs (Fig. 4a); nevertheless, the interaction frequency was higher in GJB2-positive CAFs (Fig. 4b,c). Specifically, GJB2-positive CAFs received more signals from other cells than did GJB2-negative CAFs. Interestingly, although GJB2-positive CAFs mainly signaled to cancer cells (Supplementary Fig. S4), the main source cells that actively signaled to GJB2-positive CAFs were blood vessel endothelial cells (Fig. 4c). Among all the cells that interacted with GJB2-positive CAFs, endothelial cells showed the highest frequency of signaling them (Fig. 4d). We analyzed spatial resolved transcriptome data using 10xVisium in colorectal cancer, to visualize the spatial positioning of CAFs expressing GJB2 and vascular endothelial cells (Fig. 4e). We acknowledge that this approach has limitations, however, it is worth noting that GJB2-positive cells are co-localized with spots expressing fibroblast markers such as DCN, indicating the GJB2-positive CAFs. Additionally, there is partial overlap with regions expressing vascular endothelial cell markers like CD31 (*PECAM1*). These suggest a close relationship between these two cell populations.

The main ligand–receptor pairs involved in endothelial cell–CAF interactions were found to be associated with EMC containing collagen (Fig. 4f–g and Supplementary Fig. S5). These findings suggest that endothelial cells also play an important role in fibrosis and collagen formation in the ECM through cross-talk with GJB2-positive CAFs in cancer tissues.

### GJB2 expression and clinical significance of GJB2-positive CAFs

**Figure 5:**
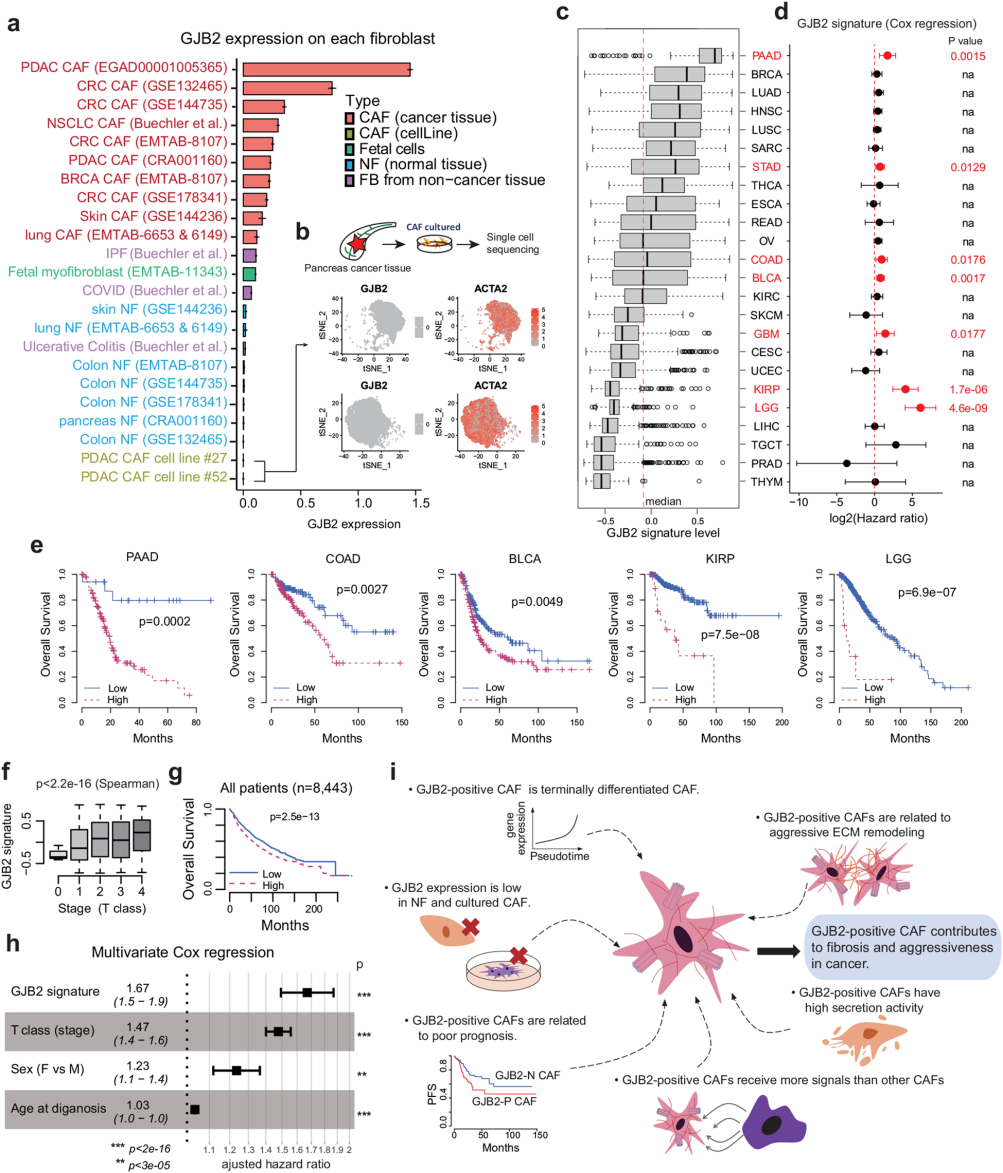
Expression and clinical significance of GJB2-positive CAFs. (**a**) Fibroblasts in various cancer tissues showed high GJB2 expression; however, GJB2 was rarely expressed in fibroblast in normal and non-cancerous tissue. (**b**) Two cultured fibroblasts from pancreatic cancer tissues showed little GJB2 expression. (**c**) GJB2-positive CAF signatures in 24 cancer types from The Cancer Genome Atlas bulk RNA-seq data. (**d**) Clinical significance of GJB2-positive CAF signatures (univariate Cox regression analysis). (**e**) Representative tumor types showing poor prognosis and a high GJB2-positive signature (log-rank test). (**f**) Tumor stage and GJB2-positive CAF signature. (**g**) Overall survival of GJB2-positive CAF signature in all patients in The Cancer Genome Atlas data (log-rank test). (**h**) Independent clinical significance of GJB2-positive CAF signature after adjustment with stage, sex, and age (multivariate Cox regression analysis). (**i**) Summary of GJB2-positive CAF characteristics. *CAF* cancer-associated fibroblast.

The expression patterns of GJB2 in various cancer and normal tissues showed that it is mainly expressed specifically in CAFs and is poorly expressed in normal tissues (Fig. 5a). Additionally, the low GJB2 expression in cultured CAFs from pancreas cancer tissues suggests that the expression of GJB2 is dependent on interactions with other cells, including vascular endothelial cells and cancer cells (Fig. 5b). Therefore, we investigated the clinical significance of GJB2-positive CAFs in cancer by measuring the GJB2 signature score in 8469 cancer tissues from The Cancer Genome Atlas (Fig. 5c and Supplementary Fig. S6a). We observed a significant correlation (Supplementary Fig. S6b) between the GJB2 signature score and the relative abundance of CAFs determined using the MCPcounter^28^. In most cancer types, patients with a high GJB2 signature score tended to have poor prognosis (Fig. 5d). Among the 24 cancer types, seven (including pancreatic adenocarcinoma (PAAD), stomach adenocarcinoma (STAD), colon adenocarcinoma (COAD), bladder urothelial carcinoma (BLCA), glioblastoma (GBM), kidney renal papillary cell carcinoma (KIRP), and brain lower-grade glioma (LGG)] showed significantly poor survival in patients with high GJB2 signature scores (Fig. 5e). In all patients with available clinical information (n = 8443), the GJB2 signature score was elevated in advanced-stage cancers (Fig. 5f). Overall, patients with a high GJB2-positive signature score showed poor prognosis (Fig. 5g), and this is a prognostic factor independent of cancer stage, age, and sex (Fig. 5h).

### Summary of GJB2-positive CAF characteristics

GJB2-positive CAFs can be characterized as follows: they are terminally differentiated; the GJB2 expression in the fibroblasts is cancer-specific; cancer with a high GJB2 signature is associated with poor prognosis; GJB2-positive CAFs communicate abundantly with other cells, including vascular endothelial cells and cancer cells; and GJB2-positive CAFs show high secretory activity in collagen production and EMC remodeling (Fig. 5i).

## Discussion

In this study, we used scRNA-seq data from multiple tumor types to identify the fibroblast subgroups associated with fibrosis. We demonstrated that GJB2-positive CAFs are a unique fibroblast subset, specialized for ECM remodeling and cancer-associated fibrosis. GJB2-positive fibroblasts are rarely found in normal tissues, but their predominance in cancer tissues suggests their lineage specificity for cancer-associated fibrosis. Considering that fibrosis in cancer tissue is associated with poor prognosis, GJB2-positive CAFs are a promising target.

Pathway analysis revealed that GJB2-positive CAFs were enriched in angiogenesis as well as protein secretion and ECM-related pathways. We also identified the interactions between GJB2-positive CAFs and other cells, finding that endothelial cells were the main source of signaling to GJB2-positive CAFs, and that the ligand–receptor pairs between endothelial cells and GJB2-positive CAFs were associated with ECM containing collagen. This suggests the importance of endothelial cells, which have been overlooked in the process of fibrosis formation. These findings correspond with those of Hsu et al.^29^, who reported that dysfunctional vascular endothelium contributes to tissue fibrosis in cancer. Our study shows that endothelial cells can secrete collagen, thereby contributing to fibrosis directly as well as signaling to CAFs.

Fibrosis and desmoplasia are frequently identified in pancreatic cancer. The GJB2-positive CAFs showed the highest abundance in pancreatic cancer among the 24 cancer types from The Cancer Genome Atlas and the various scRNA-seq datasets. In particular, the poor prognosis of cancers with high GJB2-positive signatures, including pancreatic cancer, suggests that GJB2-positive CAFs play an important role in fibrosis and clinical outcomes. We showed a significant correlation between the GJB-positive CAF signature score and the relative abundance of CAFs (CAF score) determined using MCPconter program. This finding may provide further evidence of the reliability and validity of the GJB2-positive CAF signature as a robust indicator of CAF quantity. However, it is important to acknowledge that the influence of *GJB2* expression in cancer cells cannot be excluded. Additionally, the potential impact of *GJB2* mutations in cancer cells may also be considered in prognostic impact of GJB2-positive CAF signature^30^.

In this study, we used a maker panel of three genes (*DCN*, *COL3A1*, and *THY1*) to annotate fibroblasts based on the previous study^13^. Although using only three genes may have limitations in capturing the full diversity of fibroblast populations, previous studies have demonstrated that these genes including *DCN*, *COL3A1*, *THY1,* and *BGN* can serve as canonical markers for fibroblasts^5,13,15^. In our previous study, these genes were commonly expressed in various fibroblast clusters, regardless of whether they were derived from normal or cancer tissues^5^.

While tissue-specific CAFs have been identified in various studies^31^, it is also becoming evident through integrated pan-cancer research that there are fibroblasts shared among different cancer types^18,32,33^. In this study, although there are limitations in identifying tissue-specific fibroblast populations that may exist in various datasets, through integrated analysis of multiple datasets, we have identified fibroblast population that are shared across tissues. GJB2-positive CAFs belong to one of these shared populations and GJB2-positive CAFs exhibited the highest pseudotime value, indicating that they were the most differentiated among that CAF populations.

We identified the presence of several subtypes of fibroblasts not only in CAFs but also in NFs. NFs also exhibit diverse subtypes, which can vary according to age and organs. However, the various subtypes of NFs identified in this study were similar across multiple datasets. Therefore, we combined the subgroups of NFs into one group for analysis without considering their subtypes, although it may be necessary to consider NF subtypes for further studies. Nevertheless, we confirmed that GJB2 expression was almost negative across multiple subtypes of NFs.

This study primarily focuses on human fibroblasts. Upon examining the scRNA-seq data obtained form mouse samples^34,35^, it was found that gjb2 was rarely expressed in mouse tissues including fibroblast (Supplementary Fig. S7). This may indicate either as inherent characteristic of mice or a potential limitation in the communication between mouse fibroblasts and human cancer cells. Further investigation is warranted to elucidate the underlying reasons for this disparity.

Disease associated with connexin abnormalities such as GJB2 have been reported in deafness and cardiac disease^27,36^. In cancer, research has mainly focused on the expression of cancer cells^37,38^. Studies on CAFs are extremely limited. This study identifies a unique subgroup of terminally differentiated CAFs with high GJB2 expression and demonstrated their association with ECM remodeling, their interactions with endothelial cells, and their clinical significance in cancer progression. These findings provide insight into the role of GJB2-positive CAFs in cancer and may have implications for the development of targeted therapies.

### Supplementary Information

Supplementary Figures.

Supplementary Tables.

## Data Availability

All sequencing data used in this study are available in the public domain from the Gene Expression Omnibus (accession numbers GSE132465, GSE144735, GSE178341, GSE144236, and GSE223858), ArrayExpress (E-MTAB-8107, E-MTAB-6653, E-MTAB-6149, and E-MTAB-11343), the Genome Sequence Archive (CRA001160), the European Genome-phenome Archive (EGAD00001005365), and https://www.fibroxplorer.com/.

## References

[1] Calon A, Tauriello DV, Batlle E (2014). TGF-beta in CAF-mediated tumor growth and metastasis. Semin. Cancer Biol..

[2] Gascard P, Tlsty TD (2016). Carcinoma-associated fibroblasts: Orchestrating the composition of malignancy. Genes Dev..

[3] Kalluri R, Zeisberg M (2006). Fibroblasts in cancer. Nat. Rev. Cancer.

[4] Thomas D, Radhakrishnan P (2019). Tumor-stromal crosstalk in pancreatic cancer and tissue fibrosis. Mol. Cancer.

[5] Chung HC (2021). Integrated single-cell RNA sequencing analyses suggest developmental paths of cancer-associated fibroblasts with gene expression dynamics. Clin. Transl. Med..

[6] Lee KW (2022). PRRX1 is a master transcription factor of stromal fibroblasts for myofibroblastic lineage progression. Nat. Commun..

[7] Elyada E (2019). Cross-species single-cell analysis of pancreatic ductal adenocarcinoma reveals antigen-presenting cancer-associated fibroblasts. Cancer Discov..

[8] Orimo A, Weinberg RA (2007). Heterogeneity of stromal fibroblasts in tumors. Cancer Biol. Ther..

[9] Öhlund D (2017). Distinct populations of inflammatory fibroblasts and myofibroblasts in pancreatic cancer. J. Exp. Med..

[10] Kalluri R (2016). The biology and function of fibroblasts in cancer. Nat. Rev. Cancer.

[11] Chen Y, McAndrews KM, Kalluri R (2021). Clinical and therapeutic relevance of cancer-associated fibroblasts. Nat. Rev. Clin. Oncol..

[12] Sahai E (2020). A framework for advancing our understanding of cancer-associated fibroblasts. Nat. Rev. Cancer.

[13] Lee HO (2020). Lineage-dependent gene expression programs influence the immune landscape of colorectal cancer. Nat. Genet..

[14] Pelka K (2021). Spatially organized multicellular immune hubs in human colorectal cancer. Cell.

[15] Qian J (2020). A pan-cancer blueprint of the heterogeneous tumor microenvironment revealed by single-cell profiling. Cell Res..

[16] Ji AL (2020). Multimodal analysis of composition and spatial architecture in human squamous cell carcinoma. Cell.

[17] Peng J (2019). Single-cell RNA-seq highlights intra-tumoral heterogeneity and malignant progression in pancreatic ductal adenocarcinoma. Cell Res..

[18] Buechler MB (2021). Cross-tissue organization of the fibroblast lineage. Nature.

[19] Suo C (2022). Mapping the developing human immune system across organs. Science.

[20] Xu, Z.,* et al.* STOmicsDB: A database of spatial transcriptomic data. *bioRxiv*, 2022.2003. 2011.481421 (2022).

[21] Hao Y (2021). Integrated analysis of multimodal single-cell data. Cell.

[22] Aran D (2019). Reference-based analysis of lung single-cell sequencing reveals a transitional profibrotic macrophage. Nat. Immunol..

[23] Qiu X (2017). Reversed graph embedding resolves complex single-cell trajectories. Nat. Methods.

[24] Jin S (2021). Inference and analysis of cell–cell communication using Cell Chat. Nat. Commun..

[25] Innis SE, Reinaltt K, Civelek M, Anderson WD (2021). GSEAplot: A package for customizing gene set enrichment analysis in R. J. Comput. Biol..

[26] Lavie D, Ben-Shmuel A, Erez N, Scherz-Shouval R (2022). Cancer-associated fibroblasts in the single-cell era. Nat. Cancer.

[27] Sinyuk M, Mulkearns-Hubert EE, Reizes O, Lathia J (2018). Cancer connectors: Connexins, gap junctions, and communication. Front. Oncol..

[28] Becht E (2016). Estimating the population abundance of tissue-infiltrating immune and stromal cell populations using gene expression. Genome Biol..

[29] Hsu T, Nguyen-Tran HH, Trojanowska M (2019). Active roles of dysfunctional vascular endothelium in fibrosis and cancer. J. Biomed. Sci..

[30] Jia Y (2023). Pan-cancer analysis of the prognostic and immunological role of GJB2: A potential target for survival and immunotherapy. Front. Oncol..

[31] Chhabra Y, Weeraratna AT (2023). Fibroblasts in cancer: Unity in heterogeneity. Cell.

[32] Foster DS (2022). Multiomic analysis reveals conservation of cancer-associated fibroblast phenotypes across species and tissue of origin. Cancer Cell.

[33] Luo H (2022). Pan-cancer single-cell analysis reveals the heterogeneity and plasticity of cancer-associated fibroblasts in the tumor microenvironment. Nat. Commun..

[34] Hosein AN (2019). Cellular heterogeneity during mouse pancreatic ductal adenocarcinoma progression at single-cell resolution. JCI Insight.

[35] McAndrews KM (2022). Identification of functional heterogeneity of carcinoma-associated fibroblasts with distinct IL6-mediated therapy resistance in pancreatic cancer. Cancer Discov..

[36] Delmar M, Laird DW, Naus CC, Nielsen MS, Verselis VK, White TW (2018). Connexins and disease. Cold Spring Harb. Perspect. Biol..

[37] Shettar A, Damineni S, Mukherjee G, Kondaiah P (2018). Gap junction β-2 expression is negatively associated with the estrogen receptor status in breast cancer tissues and is a regulator of breast tumorigenesis. Oncol. Rep..

[38] Meng S (2022). The prognostic value and biological significance of gap junction beta protein 2 (GJB2 or Cx26) in cervical cancer. Front. Oncol..

