# Supplementary material for: Intercellular cross-talk through lineage-specific gap junction of cancer-associated fibroblasts related to stromal fibrosis and prognosis
Source: Sci Rep. 2023 Aug 30;13:14230. doi: 10.1038/s41598-023-40957-1 (PMC10469165; doi:10.1038/s41598-023-40957-1)
Supplement: Supplementary file 1 — Supplementary Figures. [file 41598_2023_40957_MOESM1_ESM.pdf]

Supplementary Figure S1

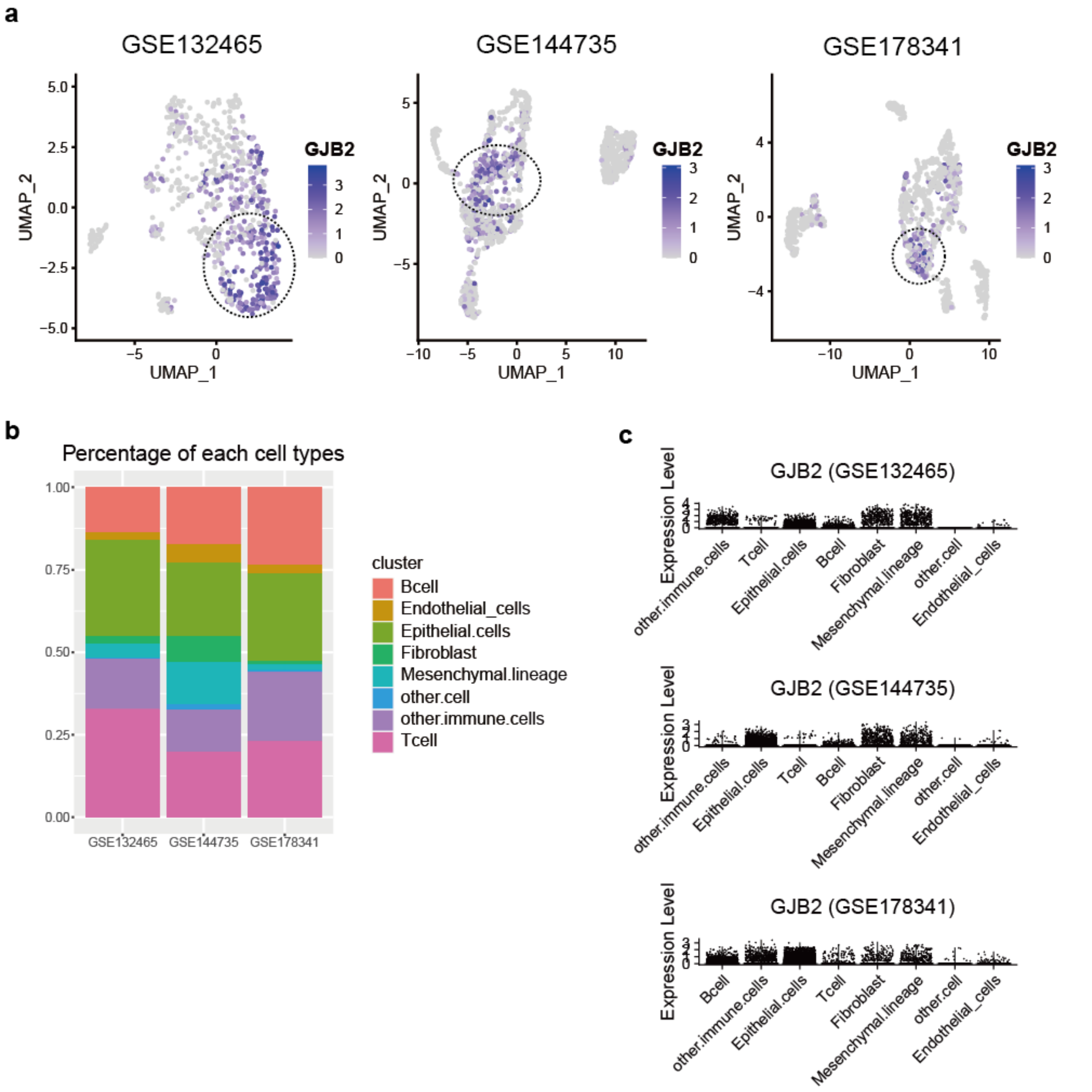

Supplementary Figure S1. (a) *GJB2* expression in a subgroups from 3 independent colorectal cancer scRNA-seq datasets. (b) Fibroblast proportion in each dataset. (c) *GJB2* expression in all cell types.

Supplementary Figure S2

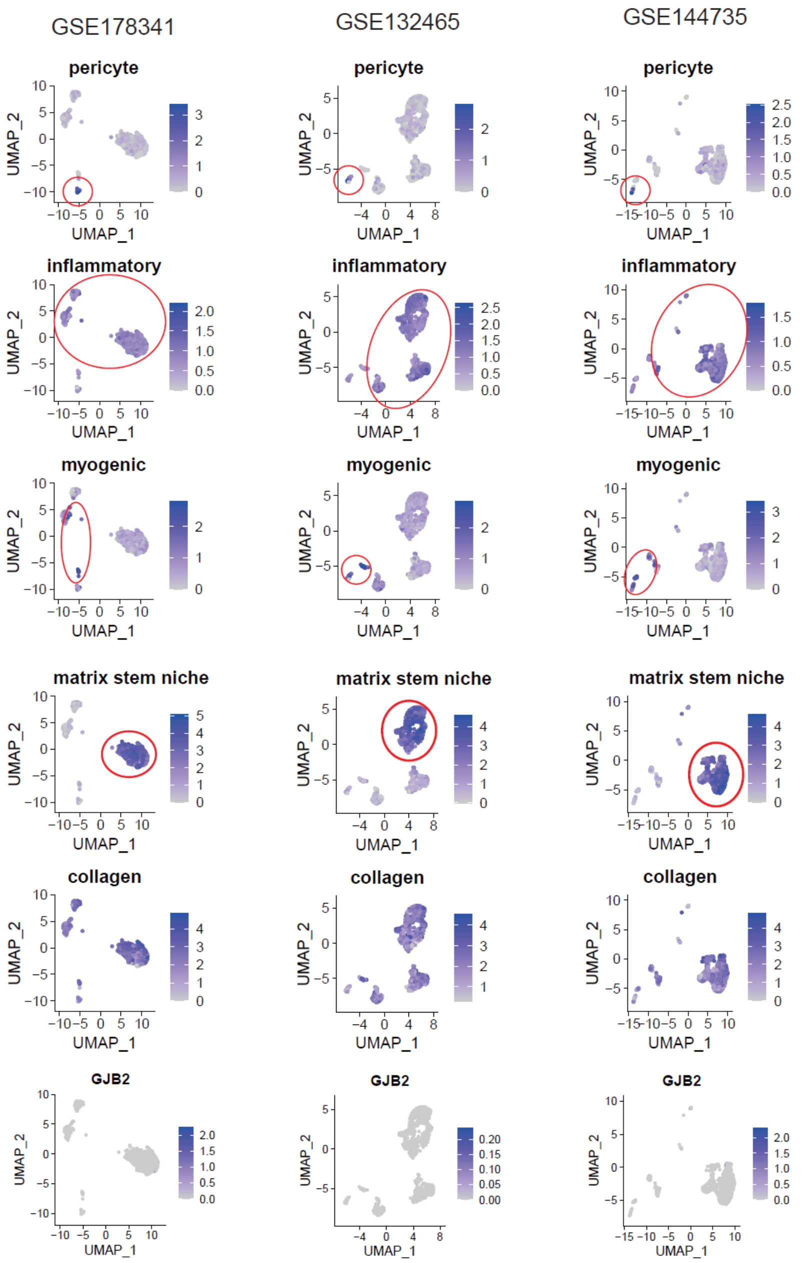

Supplementary Figure S2. Subtypes of normal fibroblasts and their expression patterns of each marker gene set (mean expression) and GJB2 expression .

Supplementary Figure S3

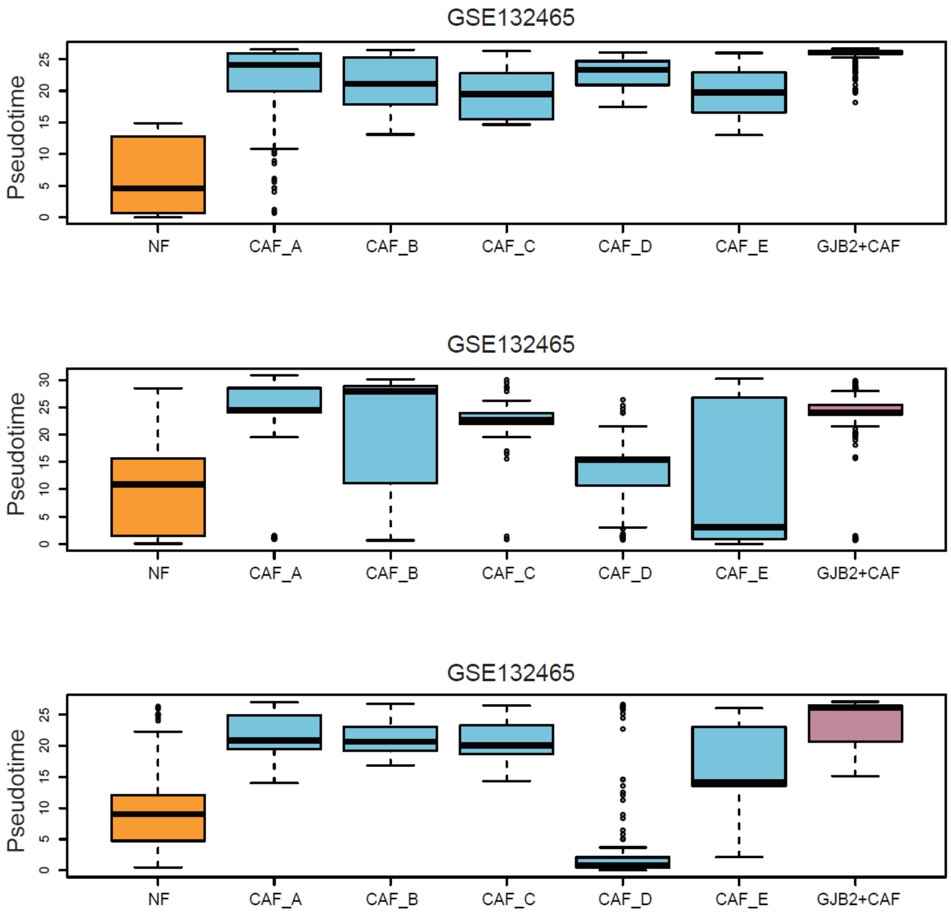

Supplementary Figure S3. Pseudotime analysis revealed that GJB2-positive CAF group showed higher level of pseudotime among CAF subgroups, which indicates that GJB2-positive CAF is terminally differentiated CAF.

Supplementary Figure S4

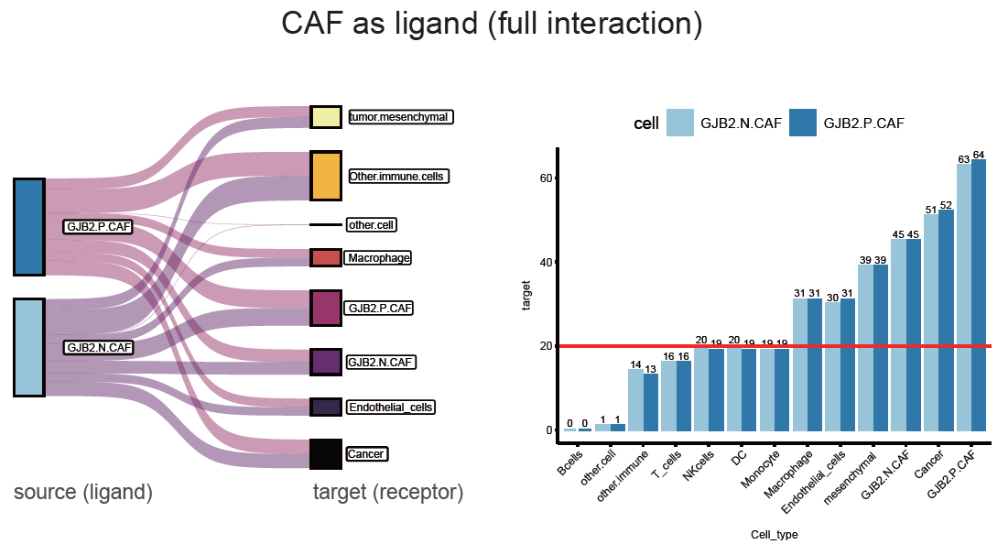

Supplementary Figure S4. Ligand and receptor interaction between CAFs and other cell types using CellChat.

Supplementary Figure S5

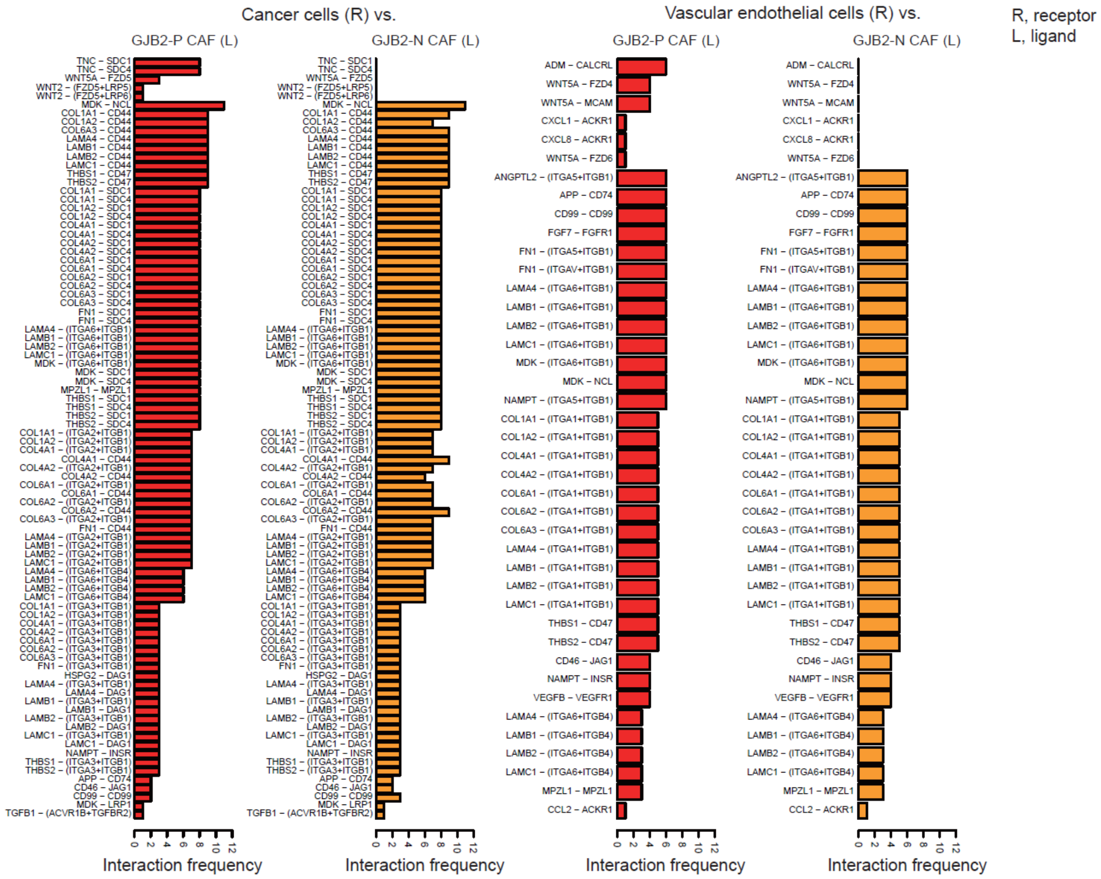

Supplementary Figure S5. Ligand and related receptor identified in GJB2-positive CAF and GJB2-negative CAF.

Supplementary Figure S6

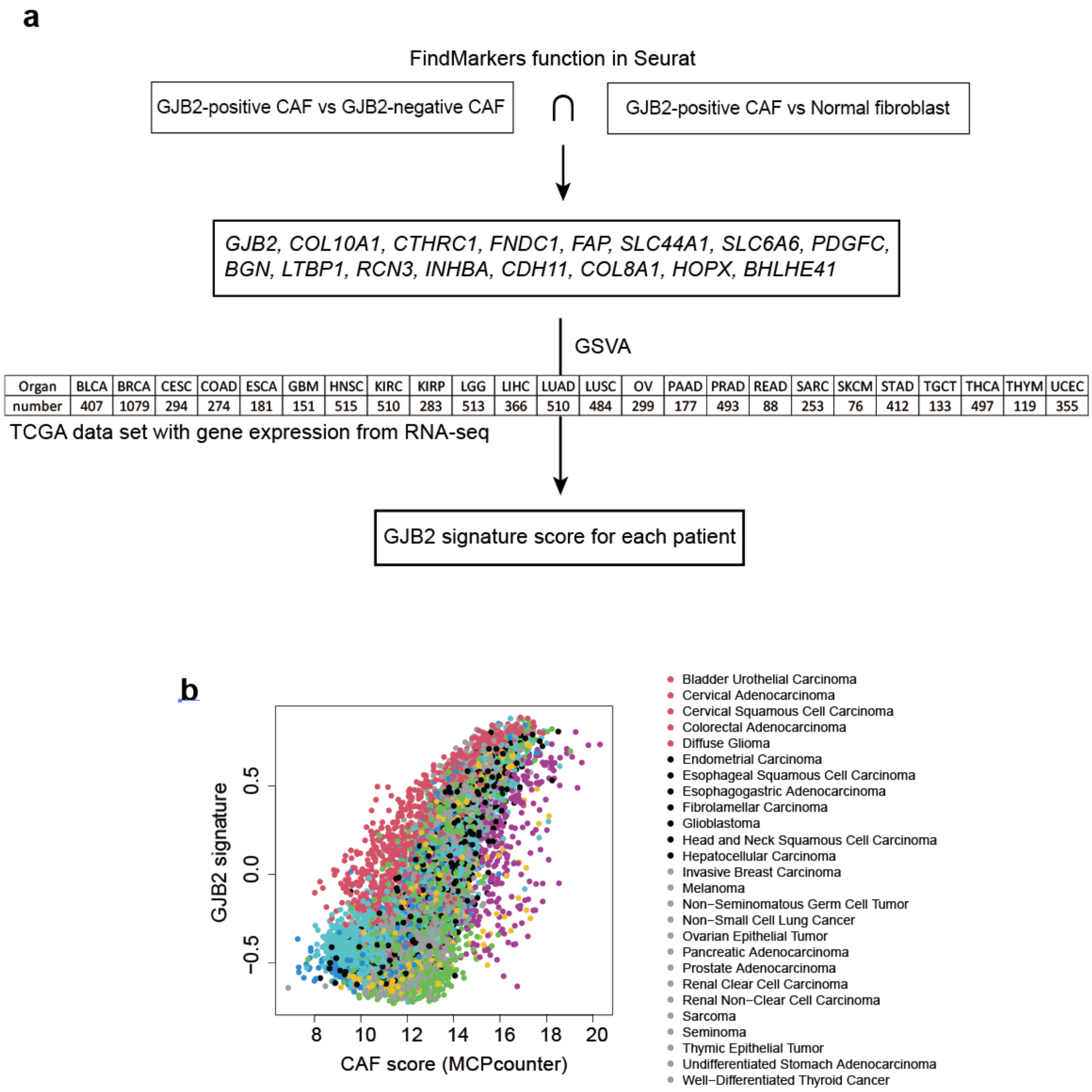

Supplementary Figure S6. (a) Identification of GJB2-positive CAF signature gene set and application of these gene set in bulk RNA sequencing data. (b) The GJB2 signature score was significantly correlated with CAF score measured by MCPcounter program in cancer tissues.

Supplementary Figure S7

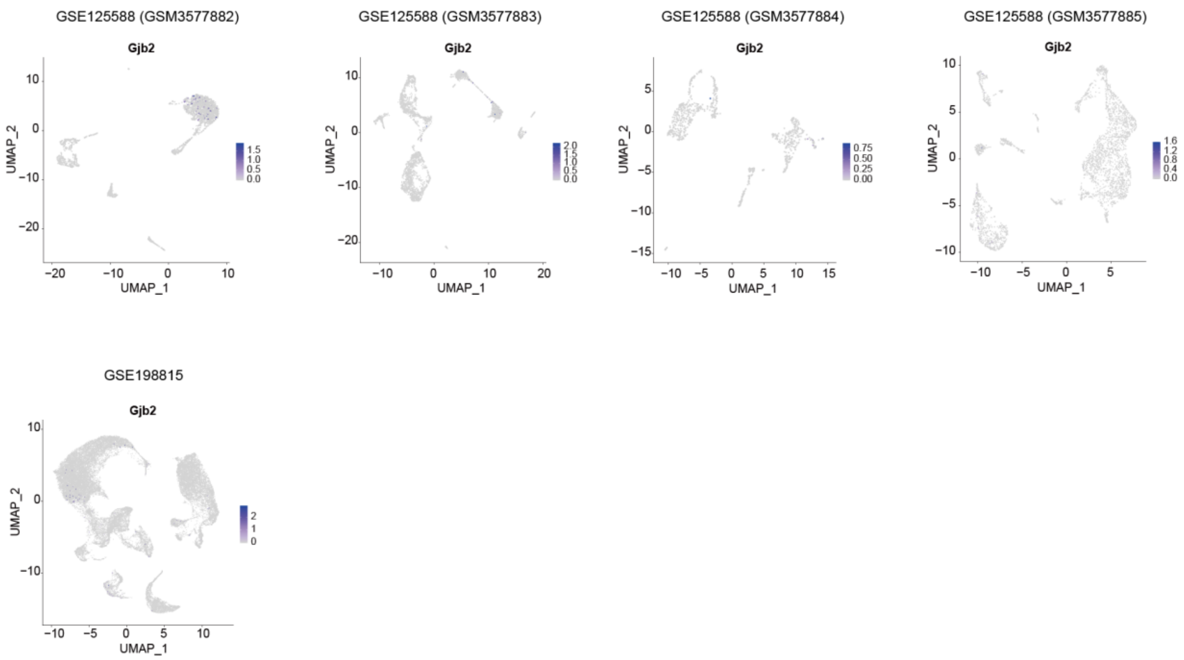

Supplementary Figure S7. Rare expression of gjb2 in mouse tissue cells including fibroblasts.
